# New Insights into Infections due to Multidrug Resistant Gram Negative Bacteria: The Interplay between Lab and Clinic

**DOI:** 10.1155/2018/8905874

**Published:** 2018-12-25

**Authors:** Alessandra Oliva, Daniele Roberto Giacobbe, Mariagrazia Di Luca, Nancy S. Miller

**Affiliations:** ^1^Department of Public Health and Infectious Diseases, Sapienza University of Rome, Rome, Italy; ^2^University of Genoa and Ospedale Policlinico San Martino – IRCCS per l'Oncologia, Genoa, Italy; ^3^Berlin-Brandenburger Centrum für Regenerative Therapien, Charité – Universitätsmedizin Berlin, Berlin, Germany; ^4^Boston Medical Center and Boston University School of Medicine, Boston, MA, USA

The rapid spread of multidrug-resistant bacteria (MDR) such as carbapenem-resistant (CR)-*Klebsiella pneumoniae *and other* Enterobacteriaceae*, CR* Acinetobacter baumannii* (CRAB), and MDR* Pseudomonas aeruginosa* has become a public health concern, especially in some countries where the diffusion of carbapenem-resistant microorganisms is endemic [1].

The knowledge of the local epidemiology and the early identification of patients at risk for MDR Gram negative (GN) acquisition/infection are crucial for prompting an appropriate empirical antimicrobial therapy, whose adequacy is a key factor for reducing mortality [2].

In the absence of randomized clinical trial data, the optimal treatment of infections caused by CR GN is a real challenge for physicians [3]. In observational studies, combination therapy seemed to bring more survival benefits than monotherapy for severe infections due to CR* Enterobacteriaceae* (CRE), whereas this is less clear for CRAB infections, with a recent randomized clinical trial suggesting no advantages of colistin-meropenem combination vs. colistin monotherapy for severe CRAB infections [4]. New agents targeting MDR GN, showing restricted/preferential activity against certain type of carbapenemases, seem to exert potent activity, but should not be used indiscriminately, in line with antimicrobial stewardship principles [5]. For instance, while the first observational data for ceftazidime-avibactam indicate high survival rates in case of CRE infections [6], the possible development of resistance is of concern and resistance strains have already been reported from different geographic areas [7]. In case of resistance to both old and novel agents, alternative revolutionary approaches such as the combination of two carbapenems [8] [i.e., the double-carbapenem (DC) regimen] might still retain an important place in therapy, especially in patients with high risk of mortality, pan-drug resistant organisms, and lack of therapeutic options [9].

Since the choice of the best regimen for the treatment of MDR GN remains a matter of debate [3], the contribution of both microbiology and pharmacology laboratories is crucial for the optimization of the available treatments. In fact, several studies suggested a correlation between the carbapenem MICs of the MDR GN and the clinical effectiveness, with regimens containing high-dose carbapenems being possibly associated with better outcomes [3]. Furthermore, clinicians should be aware that traditional antimicrobial susceptibility reports do not longer suffice to provide optimal information. Thus, the evolution of susceptibility profiling that also includes synergy testing [10] or fast and molecular microbiology is nowadays needful [2]. By using advanced technology such as comprehensive genomic analysis, the investigation and characterization of the genetic background and horizontally transferable MDR resistance in GN might be determined. However, it is also important to note that whether or not to introduce advanced (but often also costly) technologies into the laboratory workflow is a choice that should always be carefully balanced locally, taking into account also the local availability of personnel and resources, in order to both obtain and maximize the diagnostic advantage compared to standard methods [11].

Diffusion of MDR GN has also started to affect the community, thereby putting patients at risk of developing uncomplicated but difficult-to-treat infections due to MDR GN that may severely impair their quality of life and also sometimes become life-threatening because of ineffective initial treatment.

Innovative therapeutic strategies aiming at inhibition of microbial growth or virulence factors of MDR GN, such as essential oils [12] or small molecule inhibitors, are highly attractive as they may show potential as part of antimicrobial combination against MDR GN or reduce the severity of clinical manifestations and improve antibacterial immune responses. In this regard, critical components of mucosal immune system might be involved in the prevention of infection and, if altered in the expression, might correlate with infection progression.

Given the complexity of management of MDR GN infections, which need to be tailored to the severity of patients' underlying conditions and infection, to the type of MDR GN isolates with their specific antimicrobial susceptibility profiles, and to the knowledge of the local epidemiology, a multidisciplinary approach involving clinicians, the laboratory, and clinical pharmacologists as well as an antimicrobial stewardship program is recommended.

Among the articles received in response to the call for papers and after a rigorous refereeing process, 6 papers were accepted for publication in this special issue. The articles dealt with the emerging and threatening problem of MDR in GN microorganisms from both clinical and laboratory point of view.

H. Frickmann et al. thoroughly discussed the use of advanced diagnostic point-of-care options during military operations, a peculiar setting where MDR GN may be responsible for colonization and wound infections, as well as transmission to close contacts.

In their review, D. S. Lee et al. explored and enlisted essential baseline aspects for dealing with community-acquired urinary tract infections due to* Escherichia coli *in order to prevent or at least delaying development of resistance.

F. Cancelli et al. showed that the double-carbapenem regimen might represent a valid and effective therapeutic option in patients with infections due to K. pneumoniae carbapenemase (KPC) producing CR* K. pneumoniae*, including those with bacteremic infection and more severe clinical conditions. Of note, the clinical effectiveness was maintained even in the presence of extremely high meropenem MIC.

In the study conducted by S. Alousi et al., a deep genetic analysis of a strain of OXA-48 producing* E. coli* causing bloodstream infection was performed, thus highlighting the need of comprehensive genomic characterization of MDR GN for infection control and antimicrobial stewardship purposes.

In the paper by A. B. Sheremet et al., the authors showed the ability of a new small molecule, Fluorothiazinon, inhibiting type three secretion system of some Gram-negative bacteria, to reduce bacterial load and decrease lung pathology and systemic inflammation in a mouse model of lung infection due to drug-resistant* P. aeruginosa*. The reduced mortality rate of mice treated with Fluorothiazinon strongly suggested a potential use of this molecule as a therapeutic approach for the pulmonary treatment of MDR* P. aeruginosa* infections.

The review of Y.-A. Tsou et al. deals with the role of BPIFA1 (bactericidal/permeability-increasing fold 4 containing family A, member 1), a protein of innate immune system expressed in the upper airway and nasopharyngeal region including the trachea and nasal epithelium, that exhibits a bactericidal and antibiofilm activity against both Gram-positive and Gram-negative bacteria. By binding LPS, BPIFA1 is also able to inhibit the growth of* P. aeruginosa* and* K. pneumoniae*.
